# SOI Waveguide Bragg Grating Photonic Sensor for Human Body Temperature Measurement Based on Photonic Integrated Interrogator

**DOI:** 10.3390/nano12010029

**Published:** 2021-12-23

**Authors:** Hongqiang Li, Zhixuan An, Quanhua Mao, Shasha Zuo, Wei Zhu, Shanshan Zhang, Cheng Zhang, Enbang Li, Juan Daniel Prades García

**Affiliations:** 1 Tianjin Key Laboratory of Optoelectronic Detection Technology and Systems, School of Electronics and Information Engineering, Tianjin 300387, China; 2030070833@tiangong.edu.cn (Z.A.); 2030070834@tiangong.edu.cn (Q.M.); zhangshanshan@tiangong.edu.cn (S.Z.); zhangcheng@tiangong.edu.cn (C.Z.); 2 Textile Fiber Inspection Center, Tianjin Product Quality Inspection Technology Research Institute, Tianjin 300192, China; zuoshasha2021@gmail.com (S.Z.); zhuwei2120@gmail.com (W.Z.); 3 Tianjin Key Laboratory of Optoelectronic Sensor and Sensing Network Technology, Institute of Modern Optics, Nankai University, Tianjin 300071, China; 4 Centre for Medical Radiation Physics, University of Wollongong, Wollongong, NSW 2522, Australia; enbang_li@uow.edu.au; 5 Institute of Nanoscience and Nanotechnology, University of Barcelona, 08028 Barcelona, Spain; dprades@el.ub.edu

**Keywords:** waveguide Bragg grating, photonic sensor, body temperature measurement

## Abstract

A waveguide Bragg grating (WBG) provides a flexible way for measurement, and it could even be used to measure body temperature like e-skin. We designed and compared three structures of WBG with the grating period, etching depth, and duty cycle. The two-sided WBG was fabricated. An experimental platform based on photonic integrated interrogator was set up and the experiment on the two-sided WBG was performed. Results show that the two-sided WBG can be used to measure temperature changes over the range of 35–42 °C, with a temperature measurement error of 0.1 °C. This approach has the potential to facilitate application of such a silicon-on-insulator (SOI) WBG photonic sensor to wearable technology and realize the measurement of human temperature.

## 1. Introduction

In the field of biomedicine, body temperature is an important physiological parameter. Today’s typical temperature sensors include resistance thermometers, mercury thermometers and infrared radiation detectors. Resistance thermometers can routinely measure temperatures with uncertainty of ≤0.01 °C [1], but they have poor resistance to electromagnetic interference, which can cause sensor resistance to drift over time and require calibration before use. Mercury thermometers have high accuracy but are very dangerous. Infrared radiation thermometers can achieve non-contact measurement and play a key role in dealing with large-scale infectious diseases. In addition, highly sensitive NIR operating emissive thermometry can be achieved through the synergy between NIR luminescence and thermal emission [2]. However, infrared radiation thermometers have some shortcomings in accuracy and reliability, with the majority of the differences between −2 and +1 °C [3]. Most of the sensing elements of traditional medical temperature sensors are conductors, which are greatly affected by electromagnetic interference in special occasions, such as nuclear magnetic field diagnosis. The photon-based temperature sensor adopts non-conductive silicon-on-insulator (SOI) material, which can avoid the disturbance of electric field environment to measured data and has high research value in biological and medical research and treatment fields. Proposed sensor technologies range from microscale ring resonators [4,5,6,7,8,9] to fiber Bragg gratings (FBGs) [10] and waveguide Bragg gratings (WBGs) [11]. Compared with traditional sensors that use electric signals to realize detection, sensors that use optical signals for detection have the advantages of light weight, strong anti-electromagnetic interference ability, low power consumption, wide operating-frequency range, constant performance, high speed, low loss, and low crosstalk. A WBG is easy to integrate into a chip and compatible with complementary metal oxide semiconductor (CMOS)-compatible manufacturing technology [12]. This method can greatly reduce the structure size of the waveguide to a width of several hundred nanometers and improve the temperature sensitivity [13]. In photonic integrated interrogator (PIC), WBGs are waveguide structures that can realize periodic refractive index changes, so there are many ways to realize WBG. It can also be divided into different structures according to the period, waveguide structure, and refractive index distribution of grating. For grating filters, waveguide surface grating [14] or side wall grating [15] is generally used. In contrast, two-sided WBG, which belongs to side wall grating, is currently the most used structure [16,17].

In this paper, three high-sensitivity SOI WBG photonic sensors were designed. Such SOI WBG photonic sensors had a wide temperature measurement range and high accuracy. They could continuously measure temperature changes over the range 35–42 °C to realize temperature detection in the human body. The sensor could be used in biomedical sensing, forensic investigation, microbiology research, drug screening, environmental monitoring, chemical synthesis, and other fields. Different from FBG, which required ultraviolet (UV) exposure of photosensitive materials in the fiber, WBG only needed to etch periodic geometric shapes on the surface or side of the waveguide to generate periodic effective refractive index distribution of the grating, which has the advantages of small volume and easy integration. In this paper, the sensor sensitivity of WBG was improved by designing different grating structures and parameters.

## 2. Principle, Design, and Fabrication

### 2.1. Principle

The grating structure of a WBG makes the refractive index change periodically along the direction of the waveguide and forms a reflection interface in the direction of light propagation. Reflected light exists at each interface, which is superimposed to form waveguide-mode light that travels in the opposite direction. By modulating the incident-light field periodically, light of a particular wavelength can be reflected. Only by matching the phase-matching condition of the coupled mode of the Bragg grating can the specific reflection wavelength be determined.

The Bragg grating is characterized by two main parameters: resonance wavelength (*λ_B_*) and bandwidth (Δ*λ*). For a Bragg grating with a grating period (Λ), and refractive indices of the valley (*n_a_*) and peak (*n_b_*) components of the Bragg grating, the Bragg wavelength of the grating [18] is given by

(1) $$m\lambda_{B}=2\Lambda n_{eff}$$

where *m* is the grating diffraction order. *n_eff_* = *an_a_* + *bn_b_* is an effective refractive index of the structure, with *a* and *b* denoting the grating duty cycle. When the temperature changes, the refractive index of the silicon material will also change, and the grating period will also change slightly. Any small disturbance will greatly change the coupling effect of the grating, and the reflected wavelength *λ_B_* will drift. By differentiating and simplifying both sides of the Bragg grating reflection condition equation, it can be given that

(2) $$m\frac{\Delta \lambda_{B}}{\lambda_{B}}=2\left(\xi +\alpha \right)\Delta T$$

where *ξ* is the thermal light coefficient of the material, and *α* is the thermal expansion coefficient of the material. The effective refractive index and geometry of the waveguide will vary slightly with temperature, which will cause the reflected wavelength to drift in the center of the Bragg grating for temperature measurement of the human body.

### 2.2. Three WBG Structure

**Figure 1:**
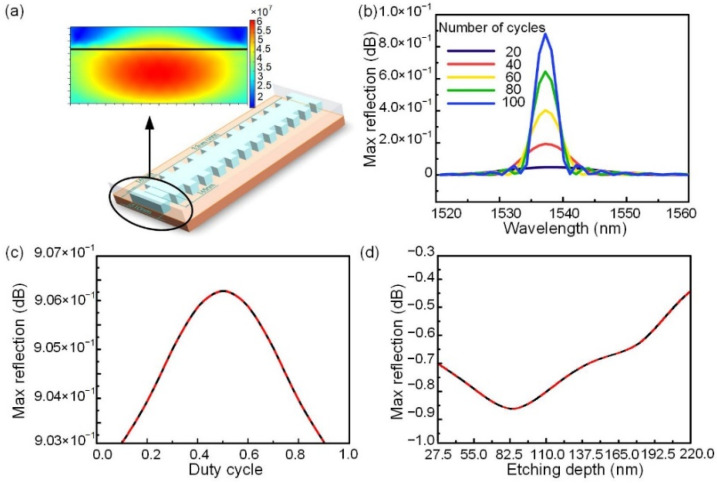
Characterization of two-sided WBG: (**a**) Schematic of two-sided WBG; (**b**) Maximum reflectivity of reflection spectrum of two-sided Bragg grating increases with number of periods; (**c**,**d**) Relationships between maximum reflectivity, and (**c**) duty cycle and (**d**) etching depth of two-sided Bragg grating.

We used the finite element method of COMSOL Multiphysics software to design and compare three structures of the temperature sensor, i.e., a top-surface WBG, one-sided WBG, and two-sided WBG, and used finite element method to characterize the performance of WBG. When modeling silicon based WBG in COMSOL Multiphysics, we added the properties of the refractive index of silicon material changing with temperature under 1550 nm incident light wave. The physical field in electromagnetic frequency domain, solid mechanical physical field, and variant geometric physical field were set by considering the thermal expansion effect of materials, and the deformation thermal conductivity of the simulator under thermal diffusion was obtained. The thermal expansion coefficient of silicon material was 2.6 × 10^−6^/k. The characterization of the two-sided WBG is shown in Figure 1. The two-sided WBG consists of 100 notches that are 0.22-µm deep and 0.51-µm wide. The grating period is 330 nm, the duty cycle 0.5, and the total length of the grating 33 µm, as shown in Figure 1a. When the number of periods of the grating is 100, the reflectivity is 0.16579, as shown in Figure 1b. The relationship between the maximum reflectivity and duty cycle of the two-sided Bragg grating is symmetrically distributed within 0.5. When the duty cycle of the grating is 0.5, the maximum reflectivity of the grating is 0.16579, as shown in Figure 1c. The maximum reflectivity increases with etching depth, as shown in Figure 1d.

**Figure 2:**
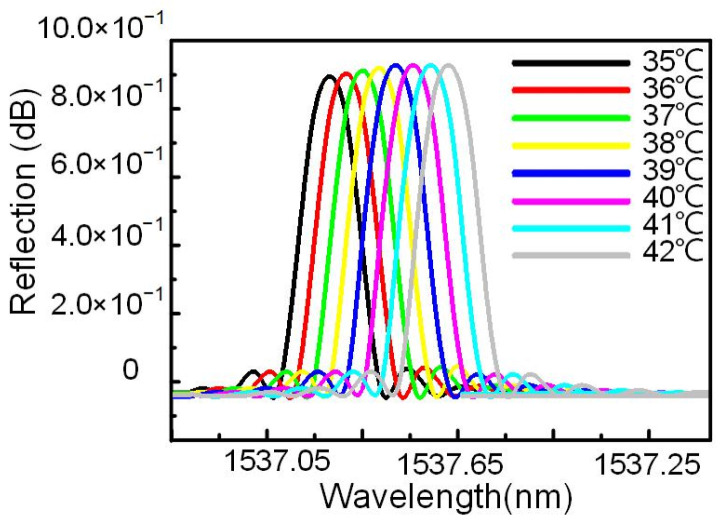
Reflection spectra of two-sided WBG at different temperatures.

The temperature of the human body is in the range of 35–42 °C, so we simulated the corresponding curve of reflection wavelength and temperature of two-sided WBG in this temperature range. (Figure 2). The central wavelength of the WBG increased with temperature. Both the linear fitting and theoretical calculated results for the temperature sensitivity of the two-sided WBG were 92 pm/°C, which indicated that the simulation results were in agreement with the theoretical calculation results. The wavelength ranged from 1537 to 1538 nm, with a maximum reflectivity of 0.910185.

**Figure 3:**
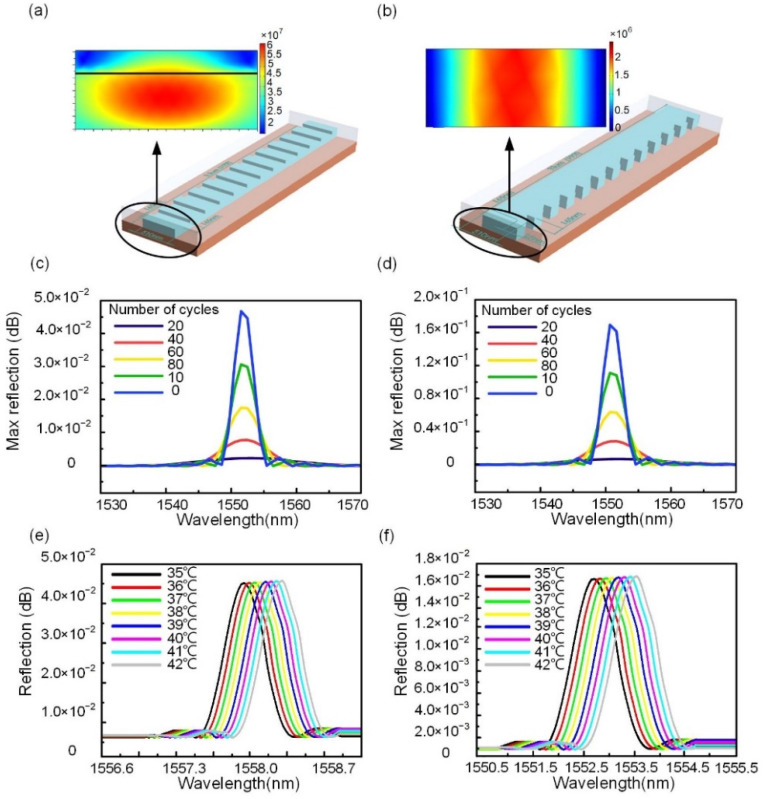
Characterization of top-surface and one-sided WBGs: (**a**) Schematic of top-surface WBG; (**b**) Schematic of one-sided WBG; (**c**) Maximum reflectivity of reflection spectrum of top-surface Bragg grating increases with number of periods; (**d**) Maximum reflectivity of reflection spectrum of one-sided Bragg grating increases with number of periods; (**e**) Reflection spectra of top-surface WBG at different temperatures; (**f**) Reflection spectra of one-sided WBG at different temperatures.

We designed two other structures by analogy, as shown in Figure 3. The top-surface WBG consisted of 100 notches that are 0.07-µm deep and 0.51-µm wide. The grating period was 330 nm; the duty cycle, 0.5; and the total length of the grating, 33 µm. The sensitivity of the temperature sensor was 76 pm/°C.

The one-sided WBG consisted of 100 notches that were 0.22-µm deep and 0.51-µm wide. The grating period was 330 nm; the duty cycle, 0.5; and the total length of the grating, 33 µm. The sensitivity of the temperature sensor was 87 pm/°C. The temperature-sensing characteristics of the WBG of the three structures are shown in Table 1.

**Figure 4:**
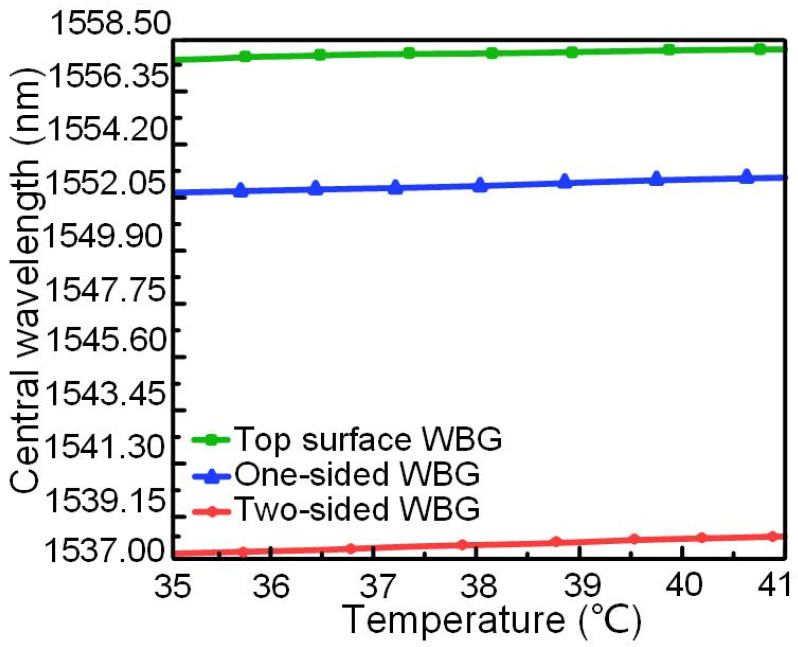
Change of WBG center wavelength with temperature in three structures.

It can be seen from Figure 4, that the central wavelength of the device with three structures is positively correlated with temperature and has an approximately linear relationship with temperature change. The data in Table 1 and Figure 4 showed the two-sided WBG had the highest sensing sensitivity and reflectivity, i.e., the best performance parameters.

Through the control variable method, the performance parameters of the top-surface, one-sided, and two-sided WBG structures were compared by etching depth and width, duty cycle, and number of cycles. The two-sided WBG, which consisted of 100 notches that were 0.22-µm deep and 0.51-µm wide, with a 0.5-duty cycle, had the best performance, with a temperature sensitivity of 92 pm/°C.

### 2.3. Device Fabrication

**Figure 5:**
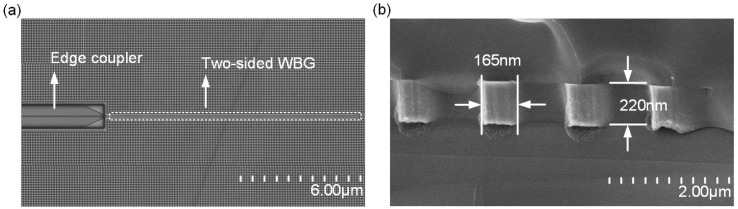
SEM image of two-sided WBG: (**a**) Top view; (**b**) Cross-sectional view.

Compared with the simulation results of the three structures, the two-sided WBG had the best performance, so we chose the two-sided WBG for process flow. The device used Belgium ISIPP50G technology, which ran on a 200-mm-thick wafer and used a 220-nm SOI layer. The period of the two-sided WBG was 330 nm; the number of cycles, 1000; the grating length, 330 μm; the duty cycle, 0.5; the full etching process reached 220 nm; the modulation depth, 25 nm; and the device size was 330 μm × 0.51 μm × 0.22 μm after the flow plate. The scanning-electron-microscope (SEM) image is shown in Figure 5.

## 3. Experimental Results

**Figure 6:**
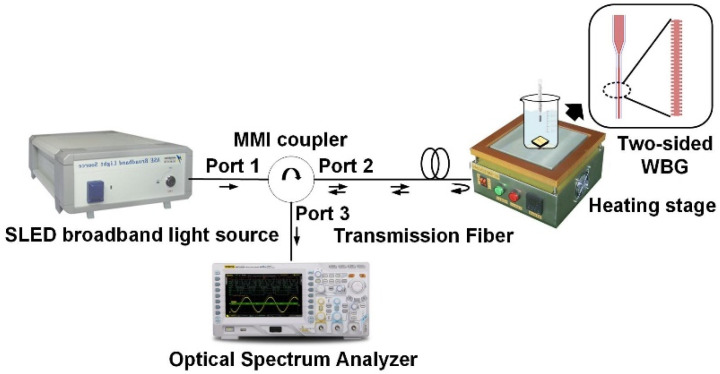
Schematic of two-sided WBG temperature sensor experimental system.

We built an experimental system for the WBG temperature sensor to test the sensitivity, maximum reflectivity, and 3-dB bandwidth of the two-sided WBG. The super-radiant light-emitting-diode (SLED) broadband light source was connected via optical fiber to port 1 of a multimode interference (MMI) coupler. Port 2 of the MMI coupler was coupled laterally to the two-sided WBG with tapered fiber optics to reduce coupling losses. The WBG will reflect the light back selectively. The light reflected from the two-sided WBG was connected to the spectrometer through three ports of the MMI coupler. The reflection wavelength and 3-dB bandwidth of the WBG was observed by a spectrograph. The two-sided WBG was fixed on the loading platform of the heating stage. The optical waveguide was then aligned with the end-face coupling platform to measure the temperature-sensing performance of the silicon-based two-sided WBG. The schematic of the experimental system is shown in Figure 6.

**Figure 7:**
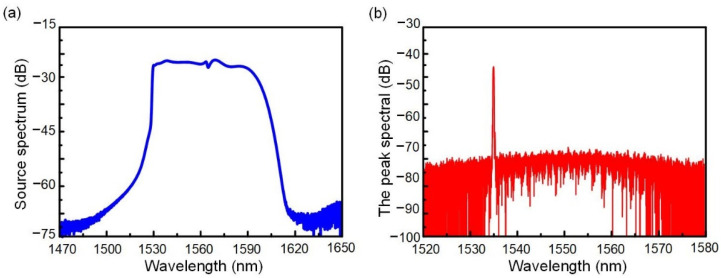
Experimental characterizations of two-sided WBG: (**a**) Output spectra of SLED broadband light source; (**b**) Temperature test results of two-sided WBG.

The spectrum of the SLED broadband light source and Bragg grating reflection spectrum at 25 °C can be seen on the spectrum analyzer, as shown in Figure 7a,b. A resonant peak with a wavelength of 1535 nm can be seen in Figure 7b, and the measured extinction ratio of the resonant peak is 13.2 dB. The insertion loss of the tapered fiber was 9.4 dB. It was seen from the results that the coupling effect of the waveguide was seen through the reflection effect of the two-sided WBG, and the waveguide had obvious wavelength selectivity.

**Figure 8:**
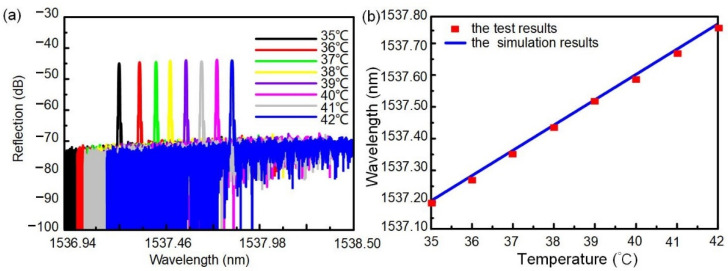
Two-sided WBG temperature sensor sensitivity test: (**a**) Two-sided WBG output spectrum at different temperatures; (**b**) Correspondence of silicon-based bilateral WBG wavelength to temperature.

The heating stage was set to 35–42 °C, and the heating interval was 1 °C. The spectrum reflected by the two-sided WBG would drift along with the temperature change, as shown in Figure 8a and Table 2. A resonant peak with a central wavelength of 1535.62 nm was observed at 35 °C, and the 3-dB bandwidth and side-mode rejection ratio of the resonant peak were 0.2296 nm and 11.7 dB, respectively. The central wavelength of the two-sided WBG redshifted and increased linearly with increasing temperature. Linear fitting was performed. The temperature-sensing sensitivity of the two-sided WBG is 80 pm/°C, and the results are shown in Figure 8b. It can be seen from the results that the device has a good wavelength-selection function and can sense the temperature of the human body.

**Figure 9:**
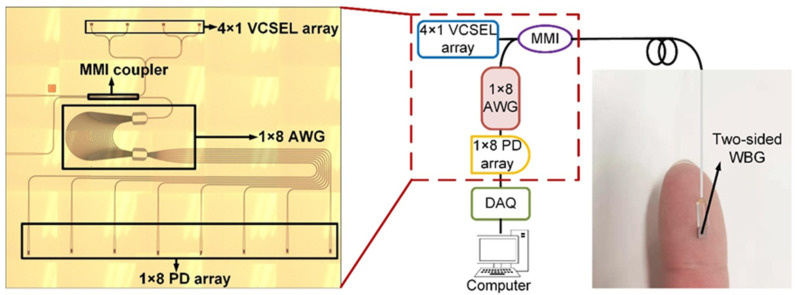
The layout and principle of the PIC of an AWG interrogation system.

The layout and principle of the PIC of an AWG interrogation is shown in Figure 9. The proposed arrayed waveguide grating (AWG) interrogator of WBG sensors consists of a PIC, data acquisition (DAQ), and a computer. The light from the long-wavelength vertical-cavity surface-emitting laser (VCSEL) array irradiates vertically to the input grating couplers, diffracts into the input waveguide of the 2 × 2 MMI coupler, and enters the two-sided WBG, the light of which is transmitted and reflected through the 2 × 2 MMI coupler and then guided into the 1 × 8 AWG. Spreading from the output waveguides of the 1 × 8 AWG, the 1 × 8 photodetector (PD) array enters through the output grating couplers, and the PD array detects the light intensity and converts it into electrical signals, which are transmitted to a computer through DAQ for data processing. We used a method to combine an edge filter with AWG to obtain the wavelength shifts of the Bragg grating [19]. The reflection spectrum shifts to the right when the external temperature of the Bragg grating increased, while the center wavelength of the VCSEL remained unchanged. The reflection spectrum changed with the effective refractive index of the Bragg grating at the center wavelength of the VCSEL, due to the energy changes in the Bragg grating reflection spectrum. Thus, the reflection spectrum power decreased during shifting of the center wavelength of the Bragg grating, due to the reduction in the effective refractive index under the center wavelength of the VCSEL. Analysis of the integral of the AWG spectrum product, the FBG reflection spectrum, and the AWG transmission spectrum over the selected detection range yields the light intensities of the AWG channels. The output light intensity can be expressed as:

(3) $$P=\left(1-L\right)\int_{0}^{\infty }I_{S}\left(\lambda \right)\cdot R_{\mathrm{WBG}}\left(\lambda \right)\cdot T_{\mathrm{AWG}}\left(m,\lambda \right)d\lambda$$

where *I_S_(λ*) is the emission spectrum of the input light, *P* is the output intensities of the channel, and *L* is the attenuation factors of the channel. The power of the reflected spectrum can be calculated in accordance with the shift of the center wavelength of the Bragg grating. The optical power can be converted into current through PD and then into voltage signal through the back-end detection circuit to obtain the relationship between the external temperature change and voltage. Owing to the differences in the center wavelength of the VCSEL, the reflection spectra of different bands of the Bragg grating exit through varying channels during the entry of multiple reflection spectra into the AWG when the VCSEL array is used. Therefore, the interrogation measurement of human body temperature under a narrowband light source using VCSELs was achieved. Table 3 shows that the experimental results of the device are basically close to the actual temperature with a temperature measurement error of less than 0.1 °C, which can sense the temperature of the human body.

## 4. Conclusions

In this paper, we compared the performance of three different WBG structures, and found that the two-sided WBG had the highest temperature sensitivity of 92 pm/°C. We then fabricated the two-sided WBG, tested the temperature sensing of the device, and compared the experimental results with the simulation results. When the input light sources were all 10 mW, the simulation result of the maximum reflectivity of two-sided WBG was 0.910185, while the tested result was only 0.56. The great difference in reflectance coefficient was due to coupling fiber loss, end-face coupling loss, and insertion loss of the end-face coupling waveguide. The sensitivity of the designed two-sided WBG temperature sensors was 92 pm/°C by simulation, and 80 pm/°C by test. The difference in sensitivity lies in the fact that the etching process led to uneven waveguide surface, unsmooth silicon and silicon coating, crystal defects, and grating-surface burrs that did not resonate well in the Bragg grating, so as to realize the reflection of resonant wavelength. The temperature interrogation system, based on the two-sided WBG, was integrated on the PIC to carry out potential wearable portable temperature measurement.

## Figures and Tables

**Table 1:** Summary of simulation results of silicon-based WBG.

WBG Type	Waveguide Width (μm)	Depth of Scan (μm)	Number of Periods (N)	Sensitivity (pm/°C)	Maximum Reflectivity (dB)
Top-surface WBG	0.51	0.07	100	76	−26.517
One-sided WBG	0.51	0.22	100	87	−15.615
Two-sided WBG	0.51	0.22	100	92	−0.856

**Table 2:** Table of characteristic parameters of WBG at different temperatures.

Temperature (°C)	35	36	37	38	39	40	41	42
**Wavelength (nm)**	1537.20	1537.28	1537.36	1537.44	1537.52	1537.60	1537.68	1537.76
**3-dB bandwidth (nm)**	0.2286	0.2307	0.21743	0.2413	0.24089	0.22780	0.2198	0.23475
**Reflectivity (dB)**	−43.5	−43.3	−43.6	−42.8	−43.4	−43.1	−43.2	−43.5

**Table 3:** Experimental results of temperature measurement in AWG interrogation PIC.

Voltage (V)	Experimental Temperature (°C)	Actual Temperature (°C)	Error (°C)
1.409138187	34.98813795	35	−0.01186
1.371939353	35.46603953	35.5	−0.03396
1.324158784	36.02021077	36	0.020211
1.274369023	36.53858574	36.5	0.038586
1.218524191	37.06137966	37	0.06138
1.165344329	37.51184258	37.5	0.011843
1.114918327	37.90343542	38	−0.09656
1.028421746	38.50914316	38.5	0.009143
0.939047644	39.06451612	39	0.064516
0.869289228	39.45798172	39.5	−0.04202
0.776814473	39.93538467	40	−0.06462
0.64463529	40.54693651	40.5	0.046937
0.521963729	41.05502975	41	0.05503
0.39150141	41.54529404	41.5	0.045294
0.278805698	41.9345401	42	−0.06546

## Data Availability

The data presented in this study are available on request from the corresponding author.
